# Hospital-based micro-elimination of hepatitis C virus: improving timely case identification and linkage to care in a tertiary hospital

**DOI:** 10.3389/fmed.2026.1876722

**Published:** 2026-07-01

**Authors:** Xiuli Wang, Lijun Chang, Fankun Kong, Yali Ma, Ying Qin

**Affiliations:** Department of Infectious Diseases, Yuncheng Central Hospital Affiliated to Shanxi Medical University, Yuncheng, Shanxi Province, China

**Keywords:** cascade of care, direct-acting antivirals, hepatitis C virus, linkage to care, micro-elimination

## Abstract

**Background:**

Despite the availability of highly effective direct-acting antivirals (DAAs), gaps in the hepatitis C virus (HCV) care cascade remain major barriers to elimination. Hospital-based micro-elimination strategies may help improve timely diagnosis and linkage to care.

**Methods:**

We retrospectively analyzed all patients who underwent anti-HCV antibody testing at Yuncheng Central Hospital between August 2021 and August 2024. Hospital-wide measures—including physician training, interdepartmental collaboration, and laboratory alert prompts for anti-HCV positive results—were implemented to improve referral and treatment. The cascade of care from screening to treatment outcomes was evaluated.

**Results:**

A total of 276,568 patients were screened for anti-HCV antibodies, identifying 4,011 seropositive individuals. Among them, 2,958 patients completed confirmatory HCV RNA testing, leading to the identification of 788 active infections. Among viremic patients, 423 (53.7%) initiated DAA therapy, all of whom completed treatment, with 418 (98.8%) achieving sustained virologic response at 12 weeks.

**Conclusion:**

Hospital-based micro-elimination strategies may help strengthen the HCV care cascade by enhancing confirmatory testing and linkage to treatment. Strengthening interdisciplinary collaboration and referral systems in tertiary hospitals may accelerate progress toward HCV elimination, particularly in high-prevalence regions.

## 1 Introduction

Hepatitis C virus (HCV) infection remains a major global public health problem and an important cause of cirrhosis and hepatocellular carcinoma. With the introduction of direct-acting antivirals (DAAs), chronic HCV infection has become a curable disease, and the World Health Organization has set the goal of eliminating viral hepatitis as a public health threat by 2030 (1–3). However, achieving this goal depends not only on treatment efficacy, but also on improving the cascade of screening, confirmatory diagnosis, referral, and treatment initiation.

In China, the prevalence of anti-HCV positivity in the general population has been estimated to be approximately 0.91%, although substantial geographic variation exists across different regions (4). Previous epidemiological studies have also suggested that HCV infection is more frequently detected among middle-aged and older adults, reflecting historical healthcare exposures and blood transfusion practices before the implementation of routine blood donor screening (5, 6). Although anti-HCV screening has been increasingly implemented in hospital settings in China, many patients with positive anti-HCV results still fail to complete HCV RNA testing or receive timely specialist referral and antiviral treatment (7, 8). This gap is especially evident in non-infectious disease departments, where awareness of standardized HCV management pathways may be limited. As a result, a substantial proportion of newly identified anti-HCV positive patients are lost before diagnosis is confirmed and treatment can begin.

Micro-elimination has been proposed as a practical strategy to accelerate HCV control by targeting specific settings or populations (9, 10). In hospital-based care, timely identification and linkage to care for newly screened anti-HCV positive patients may be particularly important, because preventing newly detected patients from being lost to follow-up is more efficient than recalling them later. Tertiary hospitals, with their large patient volume, multidisciplinary structure, and centralized laboratory systems, are well suited to implement such strategies.

Yuncheng is recognized as a high-prevalence area for hepatitis C, and Yuncheng Central Hospital, as the only tertiary care hospital in the region, receives a large and diverse patient population. Since 2021, our hospital has implemented a series of practical micro-elimination measures aimed at improving timely diagnosis, referral, and treatment of newly identified anti-HCV positive individuals. These measures included hospital-wide physician training under the guidance of the provincial Centers for Disease Control and Prevention, regular academic meetings with high-risk clinical departments, and screening alerts prompting anti-HCV positive patients to seek further evaluation in the Department of Infectious Diseases.

In the present study, we evaluated the effectiveness of these hospital-based attempts at HCV micro-elimination from August 2021 to August 2024. We further compared the characteristics of inpatient and outpatient pathways to identify the departments contributing most to new case detection and linkage to care. Our aim was to assess the feasibility and implementation outcomes of a tertiary hospital-based HCV micro-elimination model in a highly endemic region and to provide practical evidence regarding timely case identification, referral, and linkage to care in a real-world healthcare setting.

## 2 Subjects and methods

### 2.1 Study design and setting

This study was conducted at Yuncheng Central Hospital, the only tertiary care hospital in Yuncheng, Shanxi Province, China, a region with relatively high HCV prevalence. The hospital implemented a series of hospital-based micro-elimination measures beginning in 2021 to improve the identification, referral, and treatment of newly diagnosed HCV patients.

Anti-HCV testing was performed according to routine clinical practice and hospital screening protocols, including inpatient admission evaluation, pre-procedural assessment, physician-directed testing when clinically indicated, and routine outpatient screening in relevant clinical settings (11, 12). Therefore, the present study reflects a real-world hospital-based screening population rather than a predefined age-targeted screening cohort. This study was approved by the Medical Ethics Committee of Yuncheng Central Hospital, Shanxi Province (NO. YXLL-KS2026033). Informed consent was waived for all participants, due to the retrospective study design and all data were collected anonymously. All procedures in this study were in accordance with the ethical standards of the responsible committee on human experimentation and with the Helsinki Declaration of 1975, as revised in 2008.

We retrospectively analyzed data from all inpatients and outpatients who underwent anti-HCV antibody testing between August 2021 and August 2024. Patients with positive anti-HCV antibody results were further evaluated for confirmatory HCV RNA testing, specialist referral, antiviral treatment initiation, treatment completion, and sustained virologic response evaluation.

### 2.2 Micro-elimination interventions

**Figure 1:**
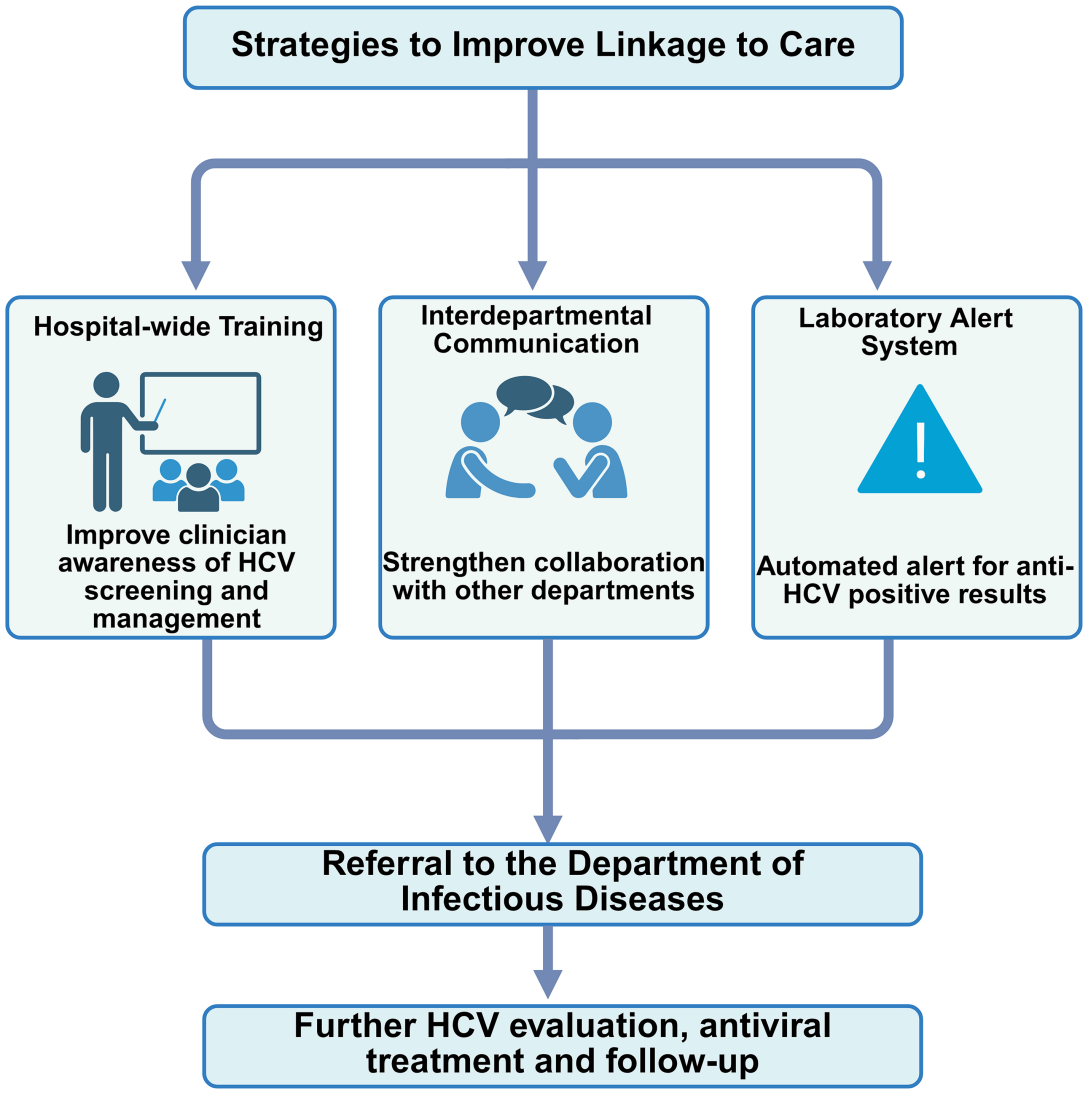
Strategies to improve linkage to care for HCV infection. Hospital-wide training, interdepartmental communication, and a laboratory-based alert system were implemented to facilitate timely referral of HCV-infected patients to the Department of Infectious Diseases for further evaluation, management, and follow-up. Created with BioRender.com.

To improve linkage to care for newly identified anti-HCV positive individuals, several interventions were implemented hospital-wide, as shown in Figure 1.

First, awareness of hepatitis C diagnosis and management was improved through hospital-wide training programs. Under the guidance of the Centers for Disease Control and Prevention (CDC), physicians from the Department of Infectious Diseases conducted educational sessions for clinicians across departments to improve understanding of HCV screening, diagnosis, and treatment pathways.

Second, interdepartmental academic communication was strengthened. Regular educational activities were organized with departments considered to have higher probabilities of encountering HCV-infected patients, including hemodialysis, oncology, gastroenterology, and other clinical departments. These activities aimed to enhance clinicians’ awareness of HCV management and facilitate timely referral of suspected cases.

Third, a laboratory-based alert system was implemented within the hospital screening system. When an anti-HCV antibody test result was positive, the laboratory information system automatically generated a highlighted alert prompting the clinician to refer the patient to the Department of Infectious Diseases for confirmatory testing and further management.

### 2.3 District-level HCV care network and intervention framework

**Figure 2:**
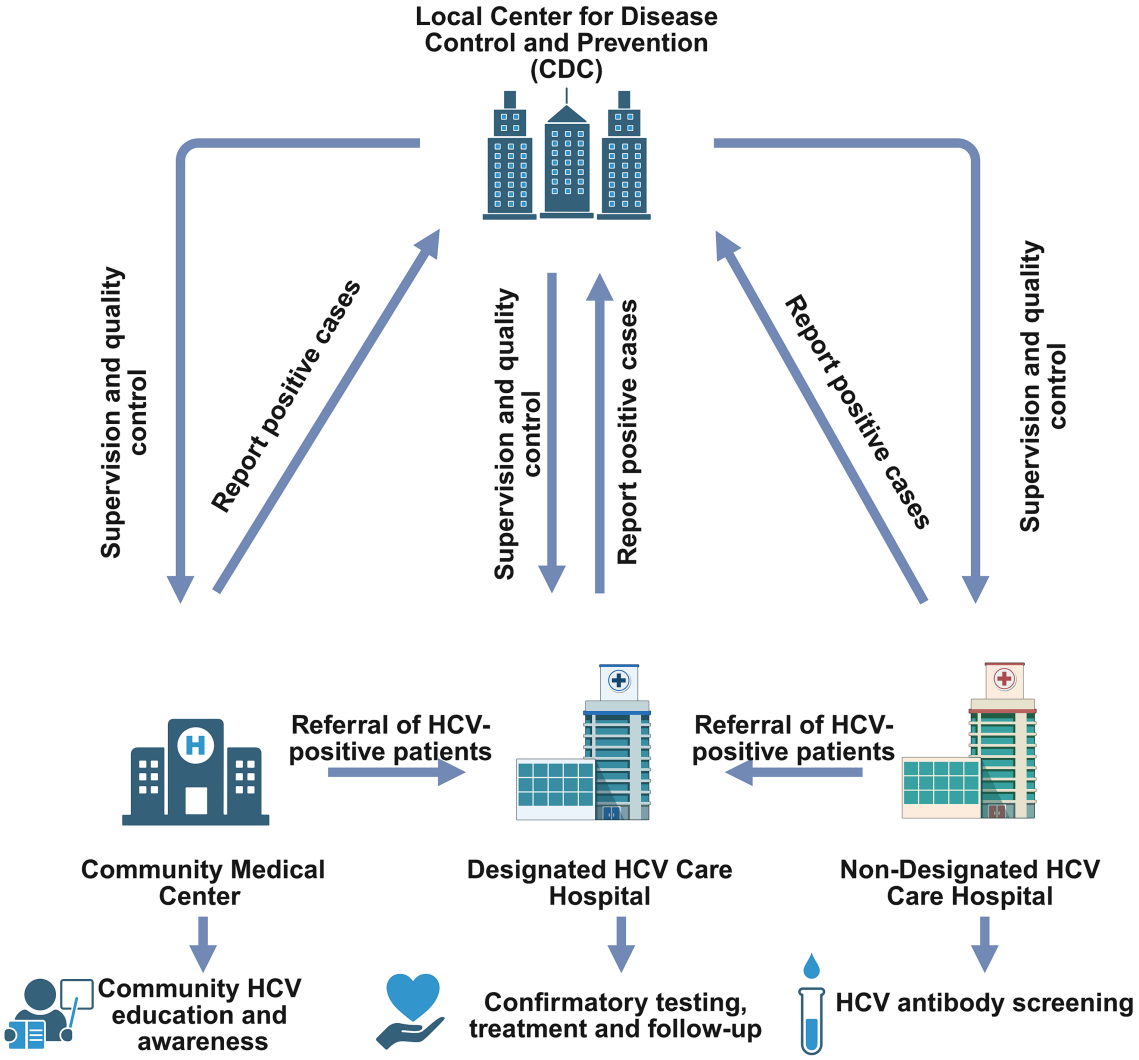
District-level HCV care network and referral framework. Community medical centers and non-designated hospitals provide HCV education and anti-HCV screening, with positive cases reported to the CDC. The CDC serves as the central coordinating body, providing supervision and quality control while facilitating referral to the designated HCV treatment hospital for confirmatory testing, antiviral treatment, and follow-up. Created with BioRender.com.

To improve the linkage to care for newly identified anti-HCV positive patients, a district-level integrated HCV care network was also established (Figure 2). This network involved community medical centers, non-designated hospitals, designated HCV treatment hospitals, and the CDC.

Community and non-designated healthcare institutions were responsible for HCV antibody screening, health education, and initial case identification. All anti-HCV positive cases were reported to the CDC, which served as the central coordinating institution for data management, supervision, and medical care quality control. The CDC also facilitated bidirectional communication and referral between healthcare institutions.

Patients identified as HCV-positive were referred to the designated treatment hospital for confirmatory testing, antiviral therapy, and standardized follow-up. This coordinated system aimed to ensure timely diagnosis, improve referral efficiency, and enhance continuity of care, thereby supporting micro-elimination of HCV at the regional level.

### 2.4 Data collection

In this study, data were retrieved from the hospital information system and laboratory database. The collected variables included demographic information, testing results for anti-HCV antibody and HCV RNA, referral to the Department of Infectious Diseases, initiation of antiviral treatment, and treatment outcomes.

Patients with positive anti-HCV antibody results were evaluated for subsequent HCV RNA testing. For those with detectable HCV RNA, antiviral treatment records and treatment outcomes were reviewed. Patients with confirmed HCV viremia received DAAs therapy according to contemporary Chinese clinical practice guidelines and routine physician assessment. Available regimens included sofosbuvir/velpatasvir, sofosbuvir plus coblopasvir, sofosbuvir plus emitasvir, and elbasvir/grazoprevir.

In addition, the numbers of patients undergoing RNA testing and the distribution of RNA-positive patients were analyzed separately for inpatient and outpatient populations, and the departments contributing to case identification were summarized.

### 2.5 Definitions

Anti-HCV antibody positivity was defined according to standard laboratory testing criteria used in the hospital laboratory. HCV RNA positivity was defined as detectable HCV RNA by polymerase chain reaction testing. Treatment initiation was defined as the prescription and administration of DAA therapy following confirmed HCV RNA positivity. Treatment completion referred to patients who completed the full antiviral treatment regimen. Sustained virologic response at 12 weeks (SVR12) was defined as undetectable HCV RNA 12 weeks after completion of antiviral therapy.

Anti-HCV antibody testing was performed using a Roche diagnostic assay. HCV RNA quantification was performed using a commercial assay from Sansure Biotech (Hunan, China), with a lower limit of detection of 25 IU/mL and an upper limit of quantification of 1 × 10^8 IU/mL. HCV RNA positivity was defined as detectable HCV RNA at or above 25 IU/mL.

For anti-HCV-positive individuals, available HCV RNA records were reviewed. Patients with a recent documented HCV RNA-negative result were not recalled for further confirmatory evaluation. However, because complete historical treatment information was not consistently available, anti-HCV-positive/HCV RNA-negative individuals could not be further classified as having spontaneous viral clearance or prior treatment-induced cure.

### 2.6 Statistical analysis

Descriptive statistical analysis was performed to summarize screening results, RNA testing rates, treatment initiation rates, and treatment outcomes. Continuous variables were expressed as means or medians where appropriate, and categorical variables were presented as frequencies and percentages. The proportions of patients undergoing HCV RNA testing, initiating treatment, completing treatment, and achieving SVR12 were calculated. All analyses were conducted using SPSS software.

## 3 Results

### 3.1 HCV screening and cascade of care

From August 2021 through August 2024, a total of 276,568 patients underwent anti-HCV antibody testing at Yuncheng Central Hospital. Among them, 4,011 (4,011/276568, 1.4%) individuals tested positive for anti-HCV antibodies.

Of these patients, 2,958 (2,958/4011, 73.7%) subsequently underwent HCV RNA testing to confirm active infection. Among those who did not complete RNA testing. The major reasons included receiving follow-up care at other medical institutions and temporary inability to attend due to personal reasons. Ultimately, 788 patients were identified with HCV RNA positive, corresponding to an RNA positivity rate of 26.6%.

Among the 788 patients with confirmed HCV viremia, 423 (53.7%) initiated DAA therapy during the study period. Of the remaining 365 patients who had not yet started treatment at our hospital, 292 (80.0%) reported plans to receive antiviral treatment in the future, 44 (12.1%) declined treatment, and 29 (7.9%) could not be contacted despite follow-up attempts.

Because this study was conducted in a real-world clinical setting, some patients may have subsequently received treatment at other medical institutions due to healthcare insurance policies, regional referral patterns, migration for employment, or personal preferences. Therefore, treatment initiation rates reported in this study may underestimate the overall proportion of patients who ultimately received antiviral therapy.

### 3.2 Outpatient pathway and implementation

**Figure 3:**
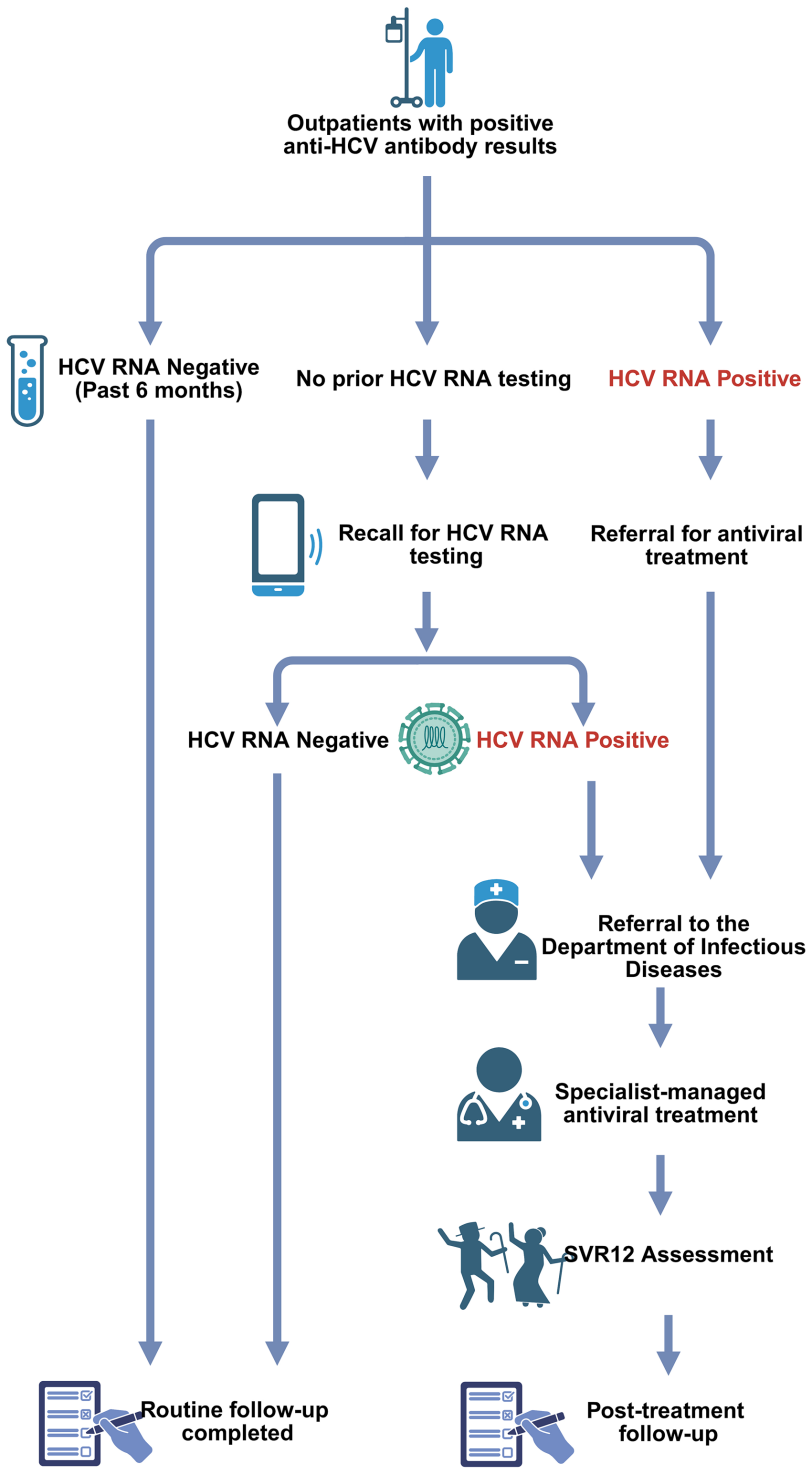
Outpatient HCV screening, recall, and treatment pathway. Anti-HCV-positive outpatients were stratified according to prior HCV RNA testing status. Patients without recent HCV RNA results were recalled for testing, whereas those with confirmed HCV RNA positivity were referred for specialist evaluation and antiviral treatment, followed by SVR12 assessment and post-treatment follow-up. Created with BioRender.com.

As shown in Figure 3, outpatients with anti-HCV positive results were stratified into three categories based on their prior RNA testing status: (1) patients with negative HCV RNA results within the past 6 months, (2) patients without available RNA results, and (3) patients with confirmed HCV RNA positivity.

Patients without recent RNA results were actively recalled for HCV RNA testing. Among those recalled, individuals were further classified as RNA negative or RNA positive. Patients with confirmed RNA positivity were referred to the Department of Infectious Diseases for specialist evaluation and antiviral treatment. Following treatment, SVR was assessed, and patients entered regular follow-up. Patients with persistently negative RNA results completed follow-up. Among the 2,958 patients who underwent HCV RNA testing, 1,659 (56.1%) were outpatients. Within this group, 300 patients were RNA positive.

### 3.3 Inpatient pathway and implementation

**Figure 4:**
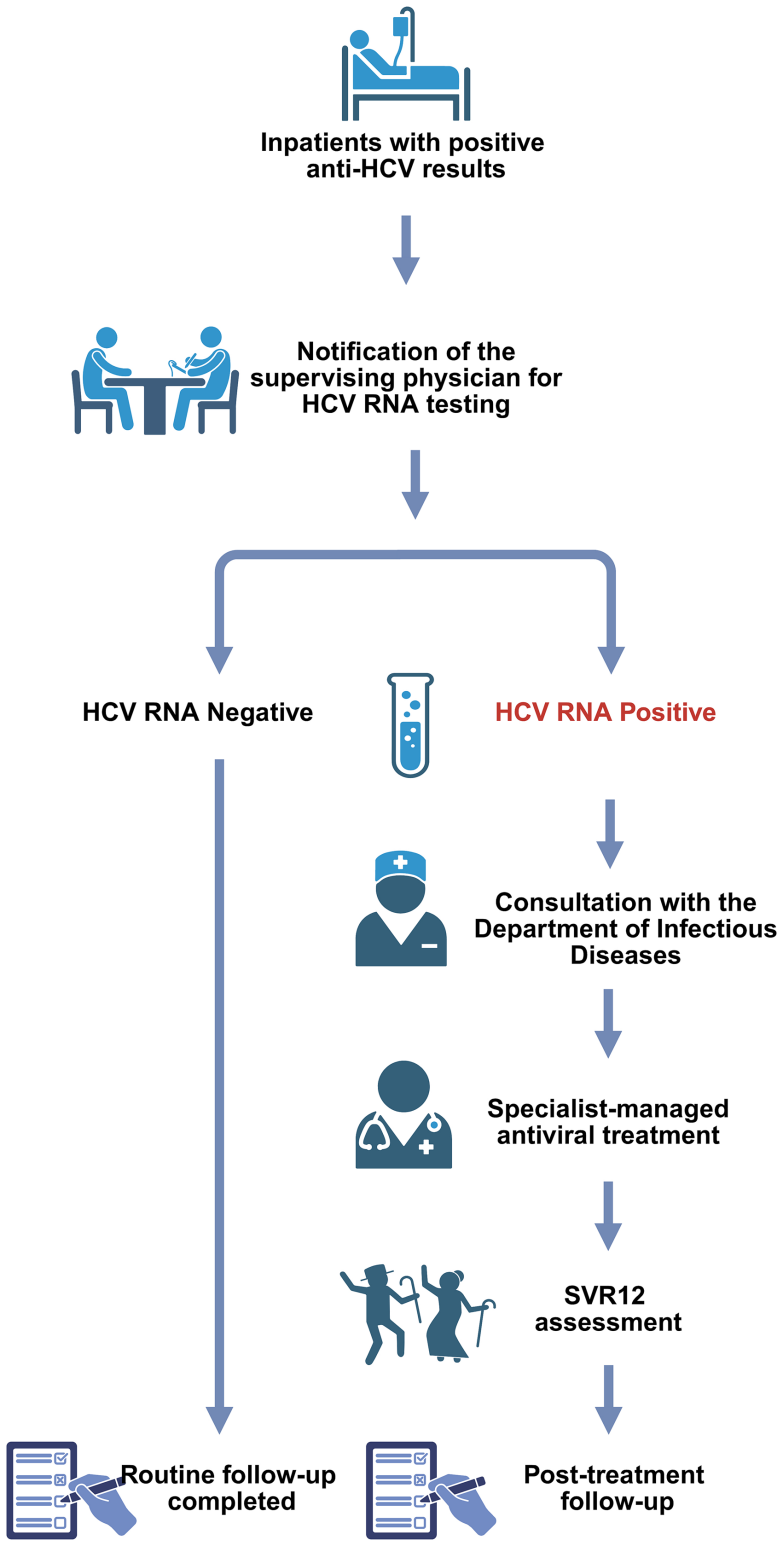
Inpatient HCV screening and treatment pathway. Anti-HCV-positive inpatients underwent physician-initiated HCV RNA testing. Patients with confirmed HCV RNA positivity received specialist consultation and antiviral treatment, followed by SVR12 assessment and post-treatment follow-up. Created with BioRender.com.

The inpatient management pathway is illustrated in Figure 4. For hospitalized patients with anti-HCV positive results, the supervising physician was directly contacted to prompt HCV RNA testing. Based on RNA results, patients were categorized as RNA negative or RNA positive.

Patients with confirmed RNA positivity received consultation from the Department of Infectious Diseases and were subsequently initiated on antiviral therapy. SVR was evaluated after treatment, followed by regular follow-up. Patients with negative RNA results completed follow-up without further intervention. Among patients undergoing RNA testing, 1,299 (43.9%) were inpatients, of whom 488 were RNA positive.

### 3.4 Departmental distribution of RNA testing and positive cases

**Figure 5:**
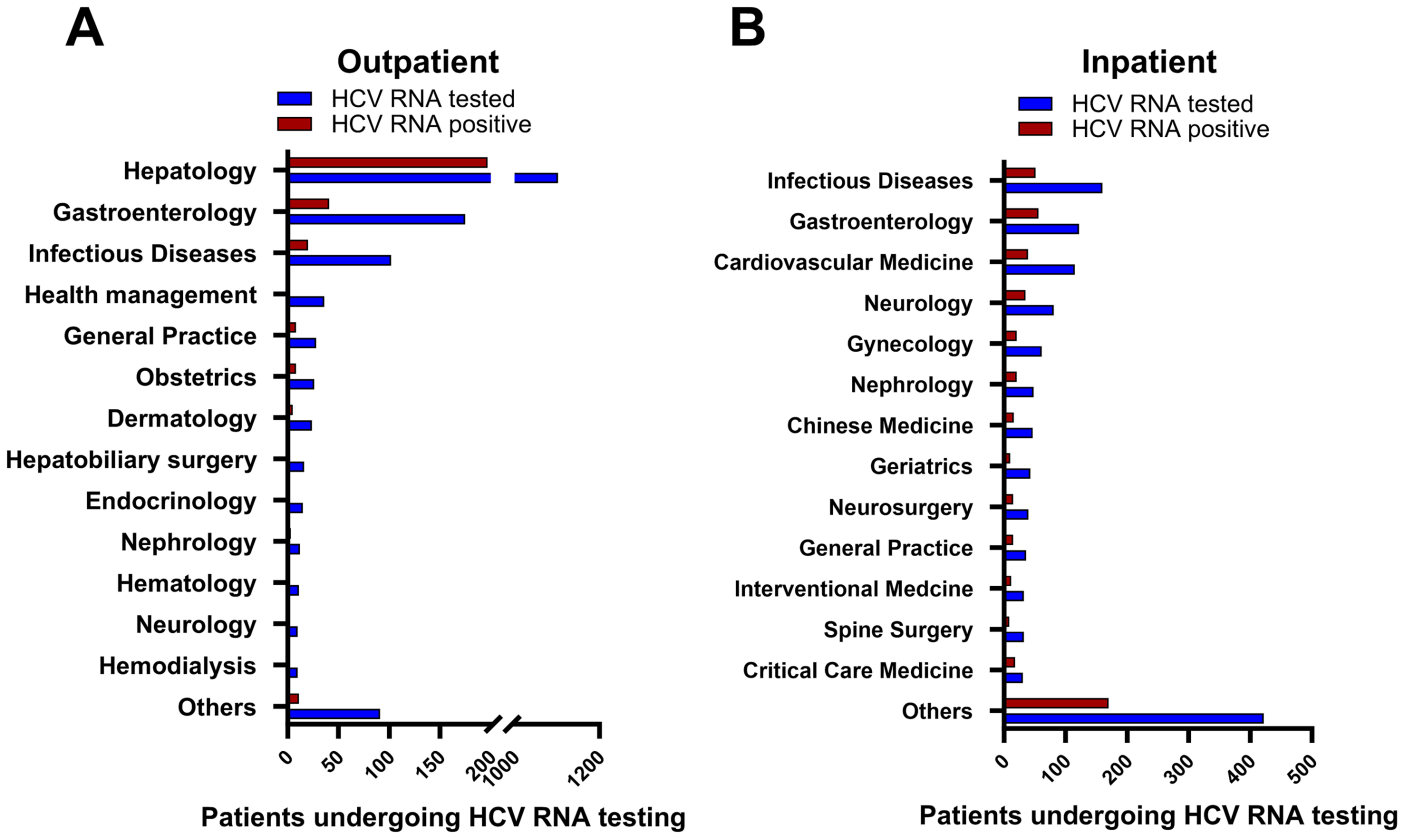
Distribution of HCV RNA testing and RNA-positive cases across clinical departments. **(A)** Outpatient setting and **(B)** inpatient setting. Blue bars represent the number of patients undergoing HCV RNA testing, and red bars represent the number of patients with confirmed HCV RNA positivity. The “Others” category includes departments with relatively small numbers of tested patients and was combined for visualization purposes. A broken x-axis was applied in panel A because of the substantially larger number of patients from the Hepatology Department.

The distribution of HCV RNA testing and HCV RNA-positive cases across clinical departments is presented in Figure 5.

Among outpatients (Figure 5A), a total of 1,659 patients underwent HCV RNA testing. The majority of tests were performed in the Hepatology Department (1,103 tests), accounting for approximately two-thirds of all outpatient RNA testing. Gastroenterology (175 tests) and Infectious Diseases (102 tests) were the next most common departments. Correspondingly, most HCV RNA-positive cases were identified in Hepatology (197 cases), followed by Gastroenterology (41 cases) and Infectious Diseases (20 cases). Additional RNA-positive patients were also detected in General Practice (8 cases), Obstetrics (8 cases), Dermatology (5 cases), Nephrology (3 cases), Neurology (2 cases), Hepatobiliary Surgery (2 cases), Hematology (1 case), and other outpatient departments. Overall, 300 HCV RNA-positive patients were identified through the outpatient pathway.

Among inpatients (Figure 5B), 488 patients were confirmed HCV RNA positive. RNA-positive patients were distributed across a broad range of clinical departments. The largest numbers of RNA-positive cases were identified in Gastroenterology (56 cases), Infectious Diseases (51 cases), Cardiovascular Medicine (39 cases), Neurology (35 cases), Nephrology (21 cases), Gynecology (21 cases), Critical Care Medicine (18 cases), Chinese Medicine (16 cases), General Practice (15 cases), Neurosurgery (15 cases), Interventional Medicine (12 cases), Geriatrics (10 cases), and Spine Surgery (9 cases), reflecting the wide distribution of HCV infection across multiple hospital departments.

### 3.5 Trends in HCV RNA testing

**Figure 6:**
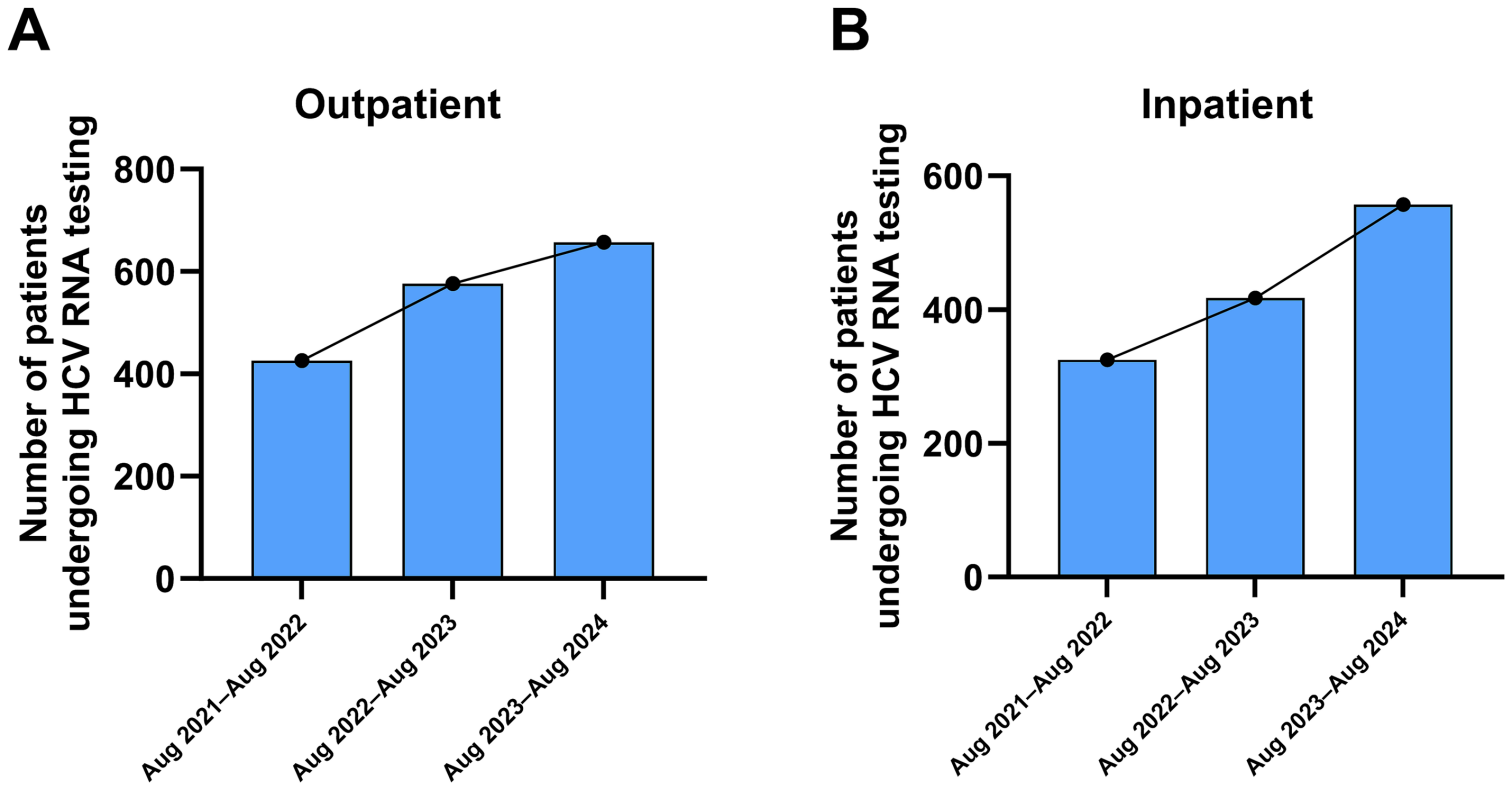
Trends in HCV RNA testing over time in outpatient and inpatient settings. **(A)** Outpatient setting and **(B)** inpatient setting. The figure illustrates the number of patients undergoing HCV RNA testing during three consecutive study periods (August 2021–August 2022, August 2022–August 2023, and August 2023–August 2024). The figure is intended to provide a descriptive illustration of temporal changes in HCV RNA testing following implementation of the hospital-based micro-elimination program.

As shown in Figure 6, the number of patients undergoing HCV RNA testing increased steadily over time in both outpatient and inpatient settings following the implementation of the micro-elimination strategy. In outpatient settings (Figure 6A), RNA testing increased progressively across the three time periods. A similar upward trend was observed among inpatients (Figure 6B), with a more pronounced increase in the later phase.

### 3.6 Treatment initiation and outcomes

All treated patients completed the prescribed treatment course. At follow-up, 418 of 423 treated patients (98.8%) achieved sustained virologic response at 12 weeks (SVR12). Overall, the care cascade in our hospital-based micro-elimination program showed relatively high rates of confirmatory RNA testing and treatment completion.

## 4 Discussion

In this study, we evaluated a series of hospital-based attempts to improve the identification and linkage to care of patients with hepatitis C virus infection in a tertiary hospital located in a highly endemic region. The anti-HCV positivity rate observed in our hospital (1.4%) was higher than the estimated prevalence reported in the general Chinese population (approximately 0.91%), which may reflect the relatively high endemicity of HCV in Yuncheng as well as the hospital-based screening population (4). Our findings demonstrated that these interventions were associated with relatively high rates of HCV RNA testing and treatment initiation, along with excellent treatment outcomes, with an SVR12 rate of 98.8% among treated patients. These results suggest that tertiary hospitals can play an important role in facilitating HCV micro-elimination by improving timely diagnosis and linkage to care.

The treatment landscape of hepatitis C has changed dramatically over the past decade. With the introduction of DAAs, sustained virologic response rates now exceed 95%, making HCV infection a curable disease (13–15). This therapeutic breakthrough has made the global elimination of hepatitis C a realistic goal. In 2016, the WHO proposed eliminating viral hepatitis as a public health threat by 2030, with key targets including diagnosing 90% of infected individuals and treating 80% of eligible patients. However, achieving these targets depends not only on treatment efficacy but also on strengthening the cascade of care, particularly the steps of screening, confirmatory diagnosis, referral, and treatment initiation. In many countries, a significant proportion of individuals who test positive for anti-HCV antibodies fail to proceed to confirmatory RNA testing or specialist care, resulting in missed opportunities for timely treatment (16–18).

China remains one of the countries with a substantial burden of chronic HCV infection (19, 20). In recent years, the Chinese government has made considerable progress in hepatitis C prevention and control, including expanding access to DAAs and launching the national “Action Plan for Eliminating Hepatitis C as a Public Health Threat (2021–2030)” (21, 22). This strategy emphasizes strengthening screening, improving referral pathways, increasing treatment coverage, and enhancing multidisciplinary collaboration. Nevertheless, previous studies have shown that although anti-HCV screening rates in tertiary hospitals are relatively high, a considerable proportion of patients do not complete subsequent diagnostic confirmation or treatment, especially when detected in non-infectious disease departments (18, 23, 24). These gaps in the care cascade remain an important barrier to achieving the national elimination targets.

Our findings highlight the importance of hospital-based interventions for improving the HCV care cascade. As the only tertiary hospital in a highly endemic region, our institution serves a large and diverse patient population. The implementation of hospital-wide training, interdepartmental communication, and laboratory alert systems helped improve clinicians’ awareness of HCV management and facilitated timely referral of anti-HCV positive patients. These measures were associated with relatively high rates of confirmatory RNA testing and treatment initiation compared with previously reported data from similar hospital settings (25–28).

Another important finding of this study is that a substantial proportion of HCV RNA-positive patients were identified outside the infectious diseases department. Departments such as gastroenterology, cardiovascular medicine, neurology, nephrology, and gynecology contributed significantly to case detection. This observation underscores the importance of hospital-wide collaboration and indicates that non-specialist departments play a critical role in identifying undiagnosed HCV infections. Strengthening awareness and standardized referral pathways across all departments may therefore be essential for preventing newly identified patients from being lost before treatment initiation.

Once patients were successfully linked to care and initiated antiviral therapy, treatment outcomes were highly favorable. Nearly all treated patients achieved SVR12, consistent with the high cure rates reported for DAA-based therapy. Because detailed follow-up information was unavailable for the small number of patients who did not achieve SVR12, the specific causes of treatment failure could not be determined. Potential explanations may include virological relapse, incomplete treatment adherence, reinfection, or incomplete post-treatment assessment. However, our findings emphasize that the major barrier to HCV elimination is no longer treatment efficacy but rather the effective identification and management of infected individuals. Improving the linkage from screening to treatment is therefore a key priority for public health programs.

Our study also provides important policy implications for HCV elimination efforts in China. While national strategies emphasize expanding screening and treatment, practical implementation at the healthcare system level remains crucial (29–32). Hospital-based micro-elimination programs can serve as effective entry points for identifying undiagnosed patients and linking them to treatment. Establishing standardized clinical pathways, strengthening collaboration between departments, and improving communication between hospital laboratories and clinicians may significantly enhance the efficiency of the HCV care cascade. In addition, integrating hospital-based programs with community healthcare systems and public health surveillance networks may further accelerate progress toward elimination.

An important implication of our findings is that progress toward HCV micro-elimination may be achievable through the coordinated implementation of existing interventions, including clinician education, interdepartmental collaboration, and laboratory-based referral alerts, without requiring complex new technologies. This may be particularly relevant in resource-limited settings where gaps in diagnosis, referral, and treatment initiation remain major barriers to care (25, 28). However, as this was a retrospective observational study conducted at a single tertiary hospital without a control group, causality cannot be established. Future prospective studies with comparative designs are needed to further evaluate the effectiveness and generalizability of hospital-based HCV micro-elimination strategies. Another issue worthy of consideration is the potential role of age-targeted screening strategies. Previous studies in China have shown that HCV infection is more prevalent among middle-aged and older adults, suggesting that age-focused screening may improve case detection efficiency and reduce screening-related costs in some settings. However, the primary objective of the present study was to evaluate a hospital-based micro-elimination strategy rather than compare alternative screening approaches. Therefore, age-specific analyses and formal cost-effectiveness evaluations were beyond the scope of this study and warrant further investigation.

Several limitations should be acknowledged. First, this was a single-center study conducted in a tertiary hospital, which may limit the generalizability of the findings. Second, although relatively high rates of HCV RNA testing and treatment initiation were observed, some RNA-positive patients did not receive antiviral therapy during the study period. Because treatment records from other institutions were unavailable, treatment uptake may have been underestimated. Third, as this study focused on evaluating a hospital-based micro-elimination program rather than a dedicated clinical cohort, detailed clinical characteristics such as HCV genotype, fibrosis stage, cirrhosis status, and comorbidities were not consistently available. Finally, time intervals from anti-HCV positivity to HCV RNA testing and from HCV RNA positivity to treatment initiation could not be reliably assessed.

## 5 Conclusion

Our findings suggest that hospital-based micro-elimination strategies are feasible and may contribute to strengthening the HCV care cascade in tertiary healthcare settings. Further studies incorporating comparative designs are needed to confirm their effectiveness and broader applicability. In our hospital, implementation of physician education, interdepartmental collaboration, and laboratory-based referral alerts was associated with relatively high rates of confirmatory RNA testing, treatment initiation, and treatment success. These findings support the potential role of tertiary hospitals in identifying previously undiagnosed HCV infections and facilitating linkage to care. Given the high efficacy of DAAs, optimizing the pathway from screening to treatment may represent an important component of HCV elimination efforts. Our experience provides real-world evidence that hospital-based micro-elimination programs may help support progress toward the WHO goal of eliminating hepatitis C as a public health threat by 2030, particularly in regions with a high burden of disease.

## Data Availability

The original contributions presented in the study are included in the article/supplementary material, further inquiries can be directed to the corresponding author.
